# Excessively increased thalamocortical connectivity and poor initial antiseizure medication response in epilepsy patients

**DOI:** 10.3389/fneur.2023.1153563

**Published:** 2023-06-16

**Authors:** Jiyuan Zhong, Ge Tan, Haijiao Wang, Yangmei Chen

**Affiliations:** ^1^International Medical College of Chongqing Medical University, Chongqing, China; ^2^Epilepsy Center, Department of Neurology, West China Hospital, Sichuan University, Chengdu, China; ^3^Department of Neurology, The Third Xiangya Hospital, Central South University, Changsha, China; ^4^Department of Neurology, The Second Affiliated Hospital of Chongqing Medical University, Chongqing, China

**Keywords:** epilepsy, antiseizure medication response, thalamus, functional connectivity, effective connectivity

## Abstract

**Objectives:**

The network mechanism underlying the initial response to antiseizure medication in epilepsy has not been revealed yet. Given the central role of the thalamus in the brain network, we conducted a case-control study to investigate the association between thalamic connectivity and medication response.

**Methods:**

We recruited 39 patients with newly diagnosed and medication-naïve epilepsy of genetic or unknown etiology, including 26 with a good response (GR group) and 13 with a poor response (PR group), and 26 matched healthy participants (control group). We measured the gray matter density (GMD) and the amplitude of low-frequency fluctuation (ALFF) of bilateral thalami. We then set each thalamus as the seed region of interest (ROI) to calculate voxel-wise functional connectivity (FC) and assessed ROI-wise effective connectivity (EC) between the thalamus and targeted regions.

**Results:**

We found no significant difference between groups in the GMD or ALFF of bilateral thalami. However, we observed that the FC values of several circuits connecting the left thalamus and the cortical areas, including the bilateral Rolandic operculum, the left insula, the left postcentral gyrus, the left supramarginal gyrus, and the left superior temporal gyrus, differed among groups (False Discovery Rate correction, *P* < 0.05), with a higher value in the PR group than in the GR group and/or the control group (Bonferroni correction, *P* < 0.05). Similarly, both the outflow and the inflow EC in each thalamocortical circuit were higher in the PR group than in the GR group and the control group, although these differences did not remain statistically significant after applying the Bonferroni correction (*P* < 0.05). The FC showed a positive correlation with the corresponding outflow and inflow ECs for each circuit.

**Conclusion:**

Our finding suggested that patients with stronger thalamocortical connectivity, potentially driven by both thalamic outflowing and inflowing information, may be more likely to respond poorly to initial antiseizure medication.

## Introduction

Approximately 47% of patients with newly diagnosed epilepsy could achieve seizure freedom by taking the first antiseizure medication (ASM) (1). It is widely acknowledged that the initial response to ASM serves as a valuable predictor of long-term seizure outcomes (2). Consequently, previous studies have made sustained efforts to investigate the clinical risk factors (3, 4) or biomarkers (5) associated with poor initial ASM responses.

With the advent of modern network science in the study of epileptic disease, previous investigations have explored brain circuits or network abnormalities in epilepsy patients that were previously imperceptible to the naked eye. Some of these abnormalities were found to be associated with ASM responses (6) or seizure outcomes (7–9). The human brain network can be conceptualized as a thalamocortical system (10), and the thalamus has been recognized as the “synchronizer” during the process of network synchronization that underlies seizure emergence and propagation (11). Studies have shown that damage to the thalamic nuclei or disruption of the thalamocortical circuits can prevent seizure occurrence (12), with the underlying mechanism being the inhibition of thalamocortical coupling and network synchronization (12, 13). It is worth noting that deep brain stimulation targeting the anterior thalamic nucleus has been approved by the Food and Drug Administration (FDA) as adjuvant therapy for adult refractory epilepsy in 2018. Therefore, it is reasonable to speculate that thalamocortical coupling features in epilepsy may be closely linked with the antiseizure effect of the initially prescribed medication.

The resting-state functional magnetic resonance imaging (fMRI) signal has been utilized to calculate the functional connectivity (FC) of the thalamus. Previous studies have found that extended and/or strengthened thalamic connectivity may increase the risk of seizure progression (14, 15) or seizure recurrence following treatment with ASM, vagal nerve stimulation, or surgery therapy (11, 16–19). Therefore, we hypothesized that patients with increased thalamocortical FC may be less likely to respond well to initial ASM treatment. Based on this hypothesis, the present study aimed to characterize thalamic functional connectivity in patients with newly diagnosed epilepsy and determine its association with the initial ASM responses.

## Materials and methods

### Participants

We enrolled 60 patients with newly diagnosed epilepsy at the West China Hospital of Sichuan University. Of these 60 patients, 49 were prospectively registered from October 2019 to December 2021, while 11 patients were identified from another database that prospectively registered patients with their first epileptic seizure that occurred from September 2016 to December 2019. The diagnosis of epilepsy was established using the practical definition proposed by the International League Against Epilepsy (ILAE) in 2014 (20). The inclusion criteria for patients were as follows: genetic or unknown etiology identified according to the 2017 ILAE Classification (21); absence of visually normal structure in MRI scans; no history of ASM usage; no history of intracranial trauma or surgery; no other neurological diseases; no apparent psychiatric comorbidities, developmental delays, or intellectual disabilities that were assessed roughly by a senior epileptologist through verbal communication with patients and their guardians. The exclusion criteria for patients included specific epileptic types or syndromes that are generally considered benign or malignant, e.g., benign Rolandic epilepsy, paroxysmal kinesigenic dyskinesia, or epileptic encephalopathy; the presence of a tumor or other systemic diseases; intolerance to MRI scanning or refusal to participate; long-term alcohol or drug abuse; and pregnancy.

Registered patients received proper medication and were regularly followed up. As of October 2022, patients who had completed a minimum of 12-month follow-up were assigned to the good and poor response groups, i.e., the GR group and the PR group, respectively, based on the definition of ASM responsiveness proposed by the ILAE in 2010 (22). Specifically, patients who achieved immediate seizure freedom following ASM treatment and remained so for at least 12 months or for a minimum of three times the longest preintervention inter-seizure interval (whichever was longer) based on seizures that occurred within the past 12 months were assigned to the GR group; those who experienced seizure recurrence during the required follow-up period were assigned to the PR group; and those who did not have a sufficient follow-up period after ASM administration with no seizure recurrence were excluded.

Given the smaller number of patients with a poor ASM response relative to those with a good response, we first identified patients in the PR group, determined age- and sex-matched patients in the GR group, and then matched healthy participants with no macroscopic MRI abnormality in the control group, using a ratio of 1:2 for the PR group to both the GR group and the control group. The procedures for patient inclusion are illustrated in Supplementary Figure S1.

### Demographic and clinical information

Baseline information was collected, including sex, age at scanning, handedness, age at onset, disease duration defined as the time from first seizure to MRI scanning, follow-up period defined as the time from the start of ASM administration to October 2022 for patients with a good response and the time to first seizure recurrence for patients with a poor response, seizure type identified based on the ILAE operational classification in 2017 (23, 24), seizure frequency, discharge laterality, and medication type.

### Image data acquisition

Brain scanning was conducted using a 3.0 Tesla MRI system (Siemens Trio, Erlangen, Germany), which was performed before ASM administration. High-resolution T1-weighted images were obtained using a three-dimensional fast spoiled gradient echo sequence with repetition time/echo time = 1900/2.26 ms; flip angle = 90°; slice thickness = 1 mm (no gap); FOV = 256 × 256 mm^2^; voxel size = 1.0 × 1.0 × 1.0 mm^3^; and the number of slices = 176. Resting-state fMRI signals were obtained using a gradient echo type planar imaging sequence with the following parameters: repetition time/echo time = 2000/30 ms; flip angle = 90°; slice thickness = 5 mm (no gap); matrix = 64 × 64; FOV = 240 × 240 mm^2^; and voxel size = 3.75 × 3.75 × 5 mm^3^. A function run lasting 406 s contained 200 volumes, each comprising 30 axial slices. The participants were asked to stay awake, relax with closed eyes, and avoid thinking about anything during scanning. A standard birdcage head coil and foam pads were used to minimize head motion, and a built-in camera was used to monitor the status of the participants during the entire process.

### Structural image preprocessing and gray matter density

The T1-weighted images were preprocessed using the voxel-based morphometry (VBM) package implemented in the DPABI 6.0 toolbox (http://rfmri.org). Briefly, the reoriented T1 images were first segmented into the gray matter, the white matter, and the cerebrospinal fluid. Second, the obtained images were spatially normalized to the Montreal Neurological Institute (MNI) space using the Diffeomorphic Anatomical Registration through Exponentiated Lie Algebra (DARTEL) algorithm. Third, the modulated gray matter images were smoothed with an 8 mm full width at half maximus (FWHM) isotropic Gaussian kernel, of which the gray matter probability values were considered the gray matter density, i.e., GMD. Finally, the bilateral thalami, identified according to the Automated Anatomical Labeling (AAL) atlas, were set as the region of interest (ROI) masks to extract the GMD value of each thalamus.

### Functional image preprocessing

Resting-state functional MRI data were preprocessed using the DPABI 6.0 toolbox. First, DICOM data were converted to NIFTI images, with the first 10 volumes discarded. Then, the converted images were corrected for intra-volume acquisition temporal differences and head motion. Subsequently, the corrected images were wrapped into a standard MNI space using the EPI template at a 3 × 3 × 3 mm^3^ resolution and spatially smoothed with an 8 mm FWHM isotropic Gaussian kernel. Thereafter, linear trends, nuisance signals (mean global, the white matter, and the cerebrospinal fluid), and head motion parameters were regressed.

Furthermore, scrubbing was also performed for head-motion correction, with the threshold of framewise displacement (FD) derived with Power's algorithm (25) set at 0.5 (1 preceding and 2 succeeding volumes) and a cubic spline strategy adopted. Finally, temporal bandpass filtering was implemented for the residuals of regressions using a bandwidth of 0.01–0.1 Hz. Subjects with noticeable head motion or rotation were excluded.

### The amplitude of low-frequency fluctuation

As detailed in the above steps, the preprocessed time course of each voxel of the whole brain was converted to the frequency domain using a fast Fourier transform to obtain the power spectrum with the DPABI 6.0 toolbox. Next, the power's square root across the predefined frequency interval, i.e., 0.01–0.1 Hz, was computed and averaged, which was taken as the amplitude of low-frequency fluctuation (ALFF). Thereafter, the ALFF values of bilateral thalami were extracted according to the thalamic masks derived from the AAL atlas.

### Functional connectivity calculation

The DPABI 6.0 toolbox calculated the resting-state FC with a seed-based correlation approach. Briefly, the thalamic mask in each hemisphere identified by the AAL atlas was set as the seed ROI, and the temporal correlation between the averaged time course of the thalamus seed and the rest of the whole brain was obtained by calculating the Pearson correlation coefficient. Then, individual correlation coefficients were normalized using the Fisher r-to-z transformation to approximate the Gaussian distribution, and thus, an entire brain z-value map was created for each participant.

### Effective connectivity calculation

The ROI-wised effective connectivity (EC) analysis for targeted thalamic circuits was performed using the Granger causality analysis package implemented in the REST 1.8 toolbox (http://www.restfmri.net/) by calculating the bivariate coefficients between the thalamus and brain clusters whose functional connectivity showed a significant intergroup difference. Outflow indicated information from the thalamus seed to clusters, and inflow indicated information from clusters to the thalamus seed. The procedures from calculating voxel-wised FC to ROI-wised EC are illustrated in Supplementary Figure S2.

### Statistical analysis

Statistical analyses of clinical variables were conducted using SPSS (version 21.0, IBM, Chicago, IL, USA). Continuous variables are presented as either a mean value ± standard deviation or a median with an interquartile range, and categorical variables are reported as the frequency with a percentage. Intergroup differences in continuous variables were analyzed using one-way ANOVA, Student *t*-test, or Mann–Whitney *U*-test, as appropriate, while differences in category variables were assessed using Pearson's chi-squared test or Fisher's exact test.

We compared the FC maps of each thalamus seed using a one-way ANOVA with sex and age at scanning as variables. We selected clusters whose FC with the thalamus survived the False Discovery Rate (FDR) correction with a *P-*value of < 0.05 as the mask ROIs and then extracted and compared the averaged FC value in each mask between each pair of groups with a significance threshold of a *P-*value of < 0.05 after Bonferroni correction. Additionally, we separately compared the GMD and ALFF values of the thalamus and the outflow and inflow EC values of target circuits that connected the thalamus and specific brain regions between each pair of groups, using a significance threshold of a *P-*value of < 0.05 after Bonferroni correction.

We also assessed the correlations between FC and clinical variables, including age at disease onset and duration, GMD and ALFF of the thalamus, and corresponding EC using the partial correlation analysis with sex and age at scanning as covariates.

## Results

### Demographic and clinical information

Of the 60 registered patients, 56 were regularly followed up, comprising 36 with a good ASM response (i.e., GR group), 14 patients with a poor ASM response (i.e., PR group), and 6 excluded due to insufficient follow-up duration. After image quality inspection, 13 patients from the PR group and 26 age- and sex-matched patients from the GR group were finally included (Supplementary Figure S1). Of the 39 included patients, 7 were identified from the prior database, and 32 were recruited later. Their differences in demographic and clinical information have been summarized in Supplementary Table S1. Additionally, 26 age- and sex-matched healthy participants were also recruited for the control group.

All participants were right-handed. There was no significant difference in either sex (*P* = 1.00) or age at scanning (*P* = 0.85) among the control group, GR group, and PR group. Additionally, none of the clinical variables displayed a significant difference between the GR group and the PR group (Table 1), except for an extended follow-up period observed in the GR group relative to the PR group (*P* < 0.001).

**Table 1:** Demographic and clinical characteristics.

**Variables**	**Control (*n =* 26)**	**GR group (*n =* 26)**	**PR group (*n =* 13)**	***P-value***
Women, n (%)	12 (46.2%)	12 (46.2%)	6 (46.2%)	1.00
Age at scanning, y	30.81 ± 9.88	32.46 ± 11.98	32.54 ± 13.31	0.85
Age at onset, y		29.73 ± 13.40	28.77 ± 14.61	0.84
Disease duration, m^*^		8.50 (0.88 - 55.75)	19.00 (1.50 - 54.00)	0.48
Follow-up period, m^*^		31.00 (25.75 - 36.00)	6.00 (5.00 - 7.50)	< 0.001
**Etiology**, ***n*** **(%)**				0.54
Genetics		2 (7.7%)	0 (0.0%)	
Unknown		24 (92.3%)	13 (100.0%)	
**Seizure type**, ***n*** **(%)**				0.74
Focal onset		18 (69.2%)	11 (84.6%)	
Generalized onset		3 (11.5%)	1 (7.7%)	
Unknown onset		5 (19.2%)	1 (7.7%)	
**Seizure frequency**, ***n*** **(%)**				0.13
≤ ten		21 (80.8%)	7 (53.8%)	
>ten		5 (19.2%)	6 (46.2%)	
**Discharge laterality**, ***n*** **(%)**				0.64
No discharge		11 (42.3%)	4 (30.8%)	
Left discharge		3 (11.5%)	1 (7.7%)	
Right discharge		6 (23.1%)	2 (15.4%)	
Bilateral discharge		6 (23.1%)	6 (46.2%)	
**Medication type**, ***n*** **(%)**				0.85
Monotherapy of levetiracetam		18 (69.2%)	8 (61.5%)	
Monotherapy of valproate		1 (3.8%)	0 (0.0%)	
Monotherapy of oxcarbazepine		3 (11.5%)	2 (15.4%)	
Monotherapy of topiramate		1 (3.8%)	0 (0.0%)	
Polytherapy		3 (11.5%)	3 (23.1%)	

GR group, patients with good response to antiseizure medication; PR group, patients with poor response to antiseizure medication; y, year; m, month.

* Presented as median with interquartile range and compared using the Mann–Whitney U-test.

### Thalamic structural and functional state

**Figure 1:**
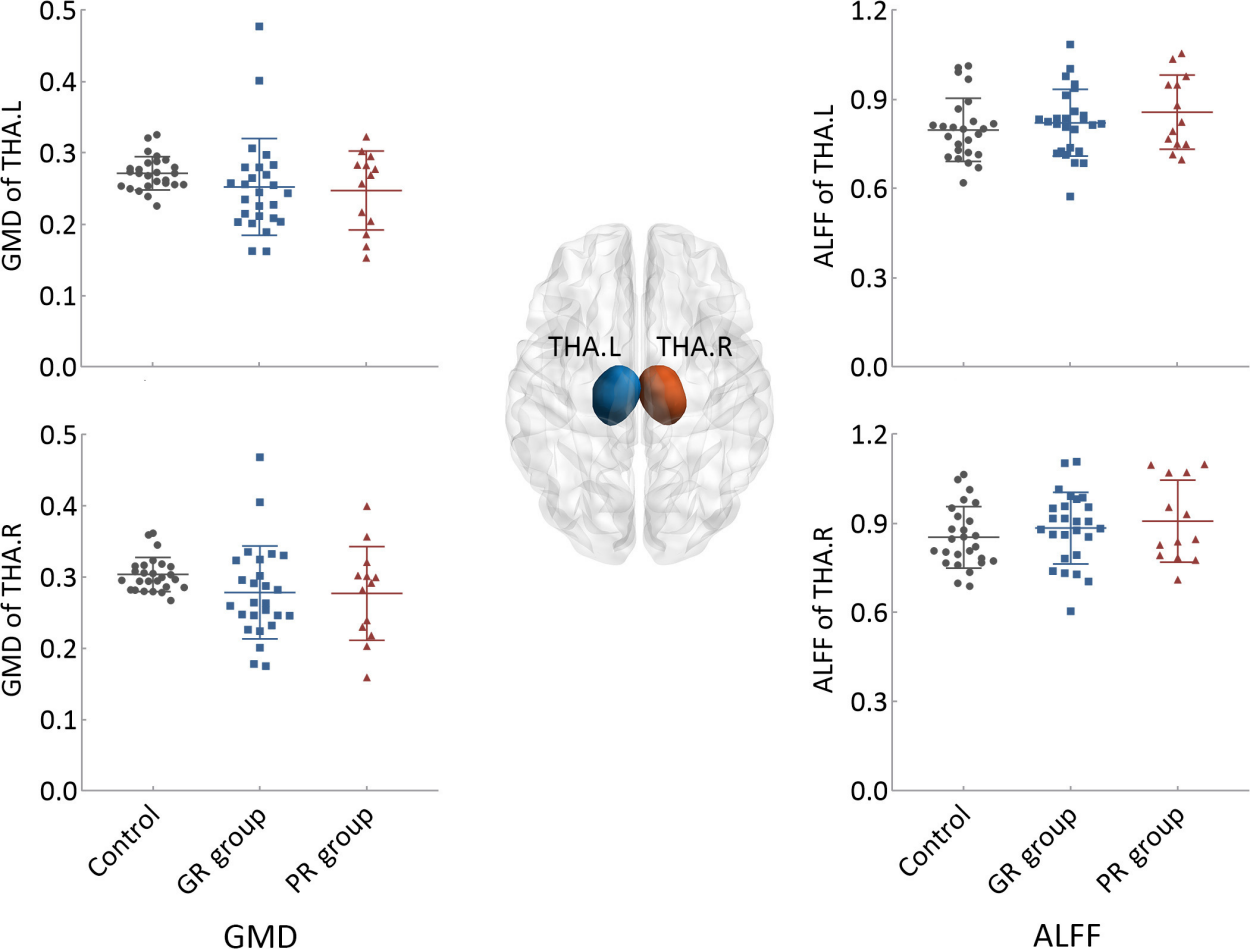
Intergroup differences in the structural and functional states of bilateral thalami. GR group, patients with good response to antiseizure medication; PR group, patients with poor response to antiseizure medication; THA.L, left thalamus; THA.R, right thalamus; GMD, gray matter density; ALFF, the amplitude of low-frequency fluctuation.

Compared to the control group, both patient groups exhibited lower GMD and higher ALFF values in the thalamus of both hemispheres, with the PR group demonstrating the most pronounced changes. However, none of these differences reached statistical significance (Figure 1).

### Thalamic functional connectivity

**Figure 2:**
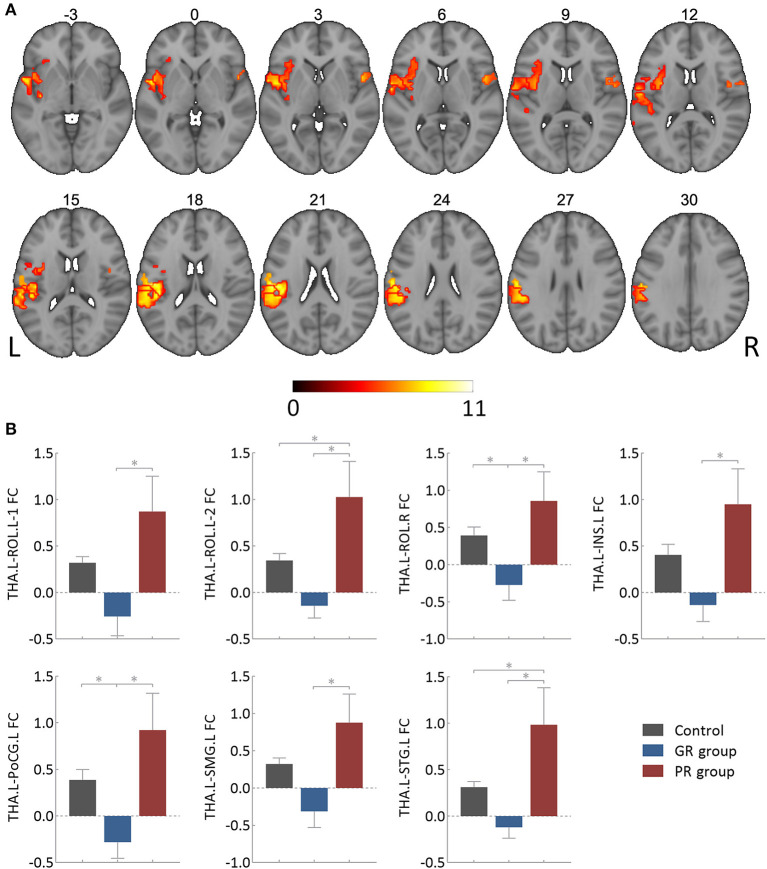
Intergroup differences in functional connectivity of the left thalamus. **(A)** Showing clusters surviving the False Discovery Rate correction (*P* < 0.05 and cluster size > 30 voxels), of which the involved brain areas were detailed in Table 1. **(B)** Showing *post hoc* analysis results of specific functional connectivity clusters. GR group, patients with good response to antiseizure medication; PR group, patients with poor response to antiseizure medication; FC, functional connectivity; THA.L, left thalamus; THA.R, right thalamus; ROL.L-1, the first cluster in the left Rolandic operculum; ROL.L-2, the second cluster in the left Rolandic operculum; ROL.R, right Rolandic operculum; INS.L, left insula; PoCG.L, left postcentral gyrus; SMG.L, left supramarginal gyrus; STG.L, left superior temporal gyrus. *Bonferroni correction (*P* < 0.05).

The results of the one-way ANOVA with FDR correction (*P* < 0.05), as illustrated in Figure 2A and Table 2, showed an intergroup difference in FC between the left thalamus and seven cortical clusters, including the bilateral Rolandic operculum (i.e., ROL.L-1, 2, and ROL.R), the left insula (INS.L), the left postcentral gyrus (PoCG.L), the left supramarginal gyrus (SMG.L) and the left superior temporal gyrus (STG.L). However, the right thalamus showed no intergroup difference in FC with any other brain regions.

**Table 2:** Intergroup differences in functional connectivity of the left thalamus.

**Clusters**	**Brain areas**	**BA**	**MNI**			**Voxels**	***F*-value**
			**X**	**Y**	**Z**		
Cluster 1	ROL.L-1	48	−51	0	3	111	8.36
Cluster 2	ROL.L-2	48	−48	−18	18	65	9.76
Cluster 3	ROL.R	48	51	6	0	60	7.60
Cluster 4	INS.L	47/48	−36	−15	−3	216	8.33
Cluster 5	PoCG.L	48	−51	−18	18	57	10.39
Cluster 6	SMG.L	2/48	−54	−21	21	155	9.58
Cluster 7	STG.L	42/48	−60	−6	6	249	9.35

Showing clusters surviving the False Discovery Rate correction with a P-value of < 0.05 and cluster size >30 voxels. BA, Brodmann area; MNI, Montreal Neurological Institute; ROL.L-1, the first cluster in left Rolandic operculum; ROL.L-2, the second cluster in the left Rolandic operculum; ROL.R, right Rolandic operculum; INS.L, left insula; PoCG.L, left postcentral gyrus; SMG.L, left supramarginal gyrus; STG.L, left superior temporal gyrus.

The results of subsequent *post-hoc* analysis are detailed in Figure 2B. All of these seven thalamocortical circuits had a lower FC value in the GR group and a higher value in the PR group compared to the control group. The PR group also had a higher FC value than the GR group for each circuit, which could survive the Bonferroni correction at a *P-*value of < 0.05.

### Thalamic effective connectivity

**Figure 3:**
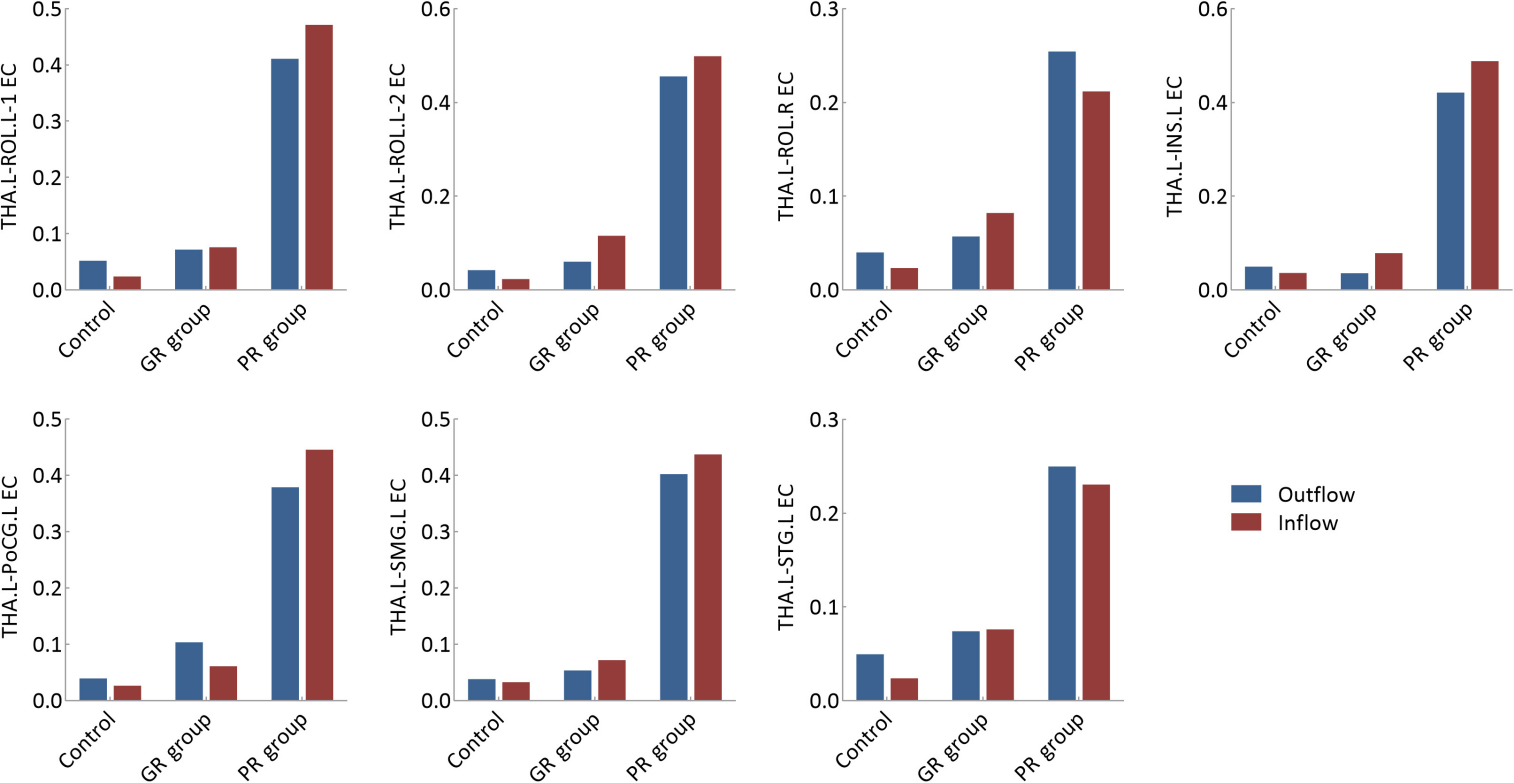
Effective connectivity analysis of specific thalamocortical circuits. GR group, patients with good response to antiseizure medication; PR group, patients with poor response to antiseizure medication; EC, effective connectivity; THA.L, left thalamus; THA.R, right thalamus; ROL.L-1, the first cluster in the left Rolandic operculum; ROL.L-2, the second cluster in the left Rolandic operculum; ROL.R, right Rolandic operculum; INS.L, left insula; PoCG.L, left postcentral gyrus; SMG.L, left supramarginal gyrus; STG.L, left superior temporal gyrus.

The EC values of each thalamocortical circuit are presented in Figure 3. The outflowing and inflowing information of each circuit in the PR group was higher than those in the control and GR groups, although none of these differences reached statistical significance after Bonferroni correction at a *P-*value of < 0.05.

### Correlation analysis

**Figure 4:**
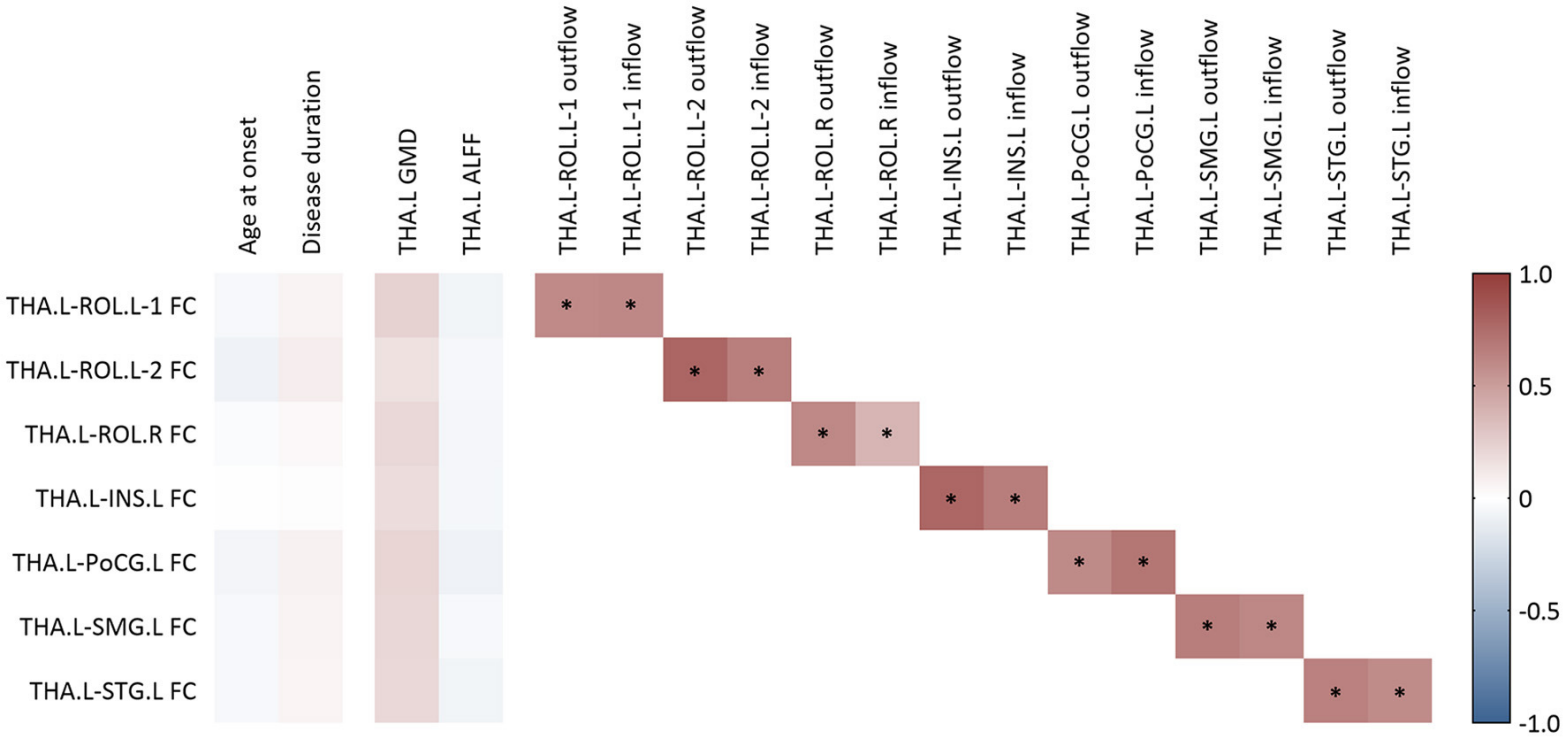
Correlation analysis for functional connectivity. FC, functional connectivity; GMD, gray matter density; ALFF, amplitude of low-frequency fluctuation; THA.L, left thalamus; THA.R, right thalamus; ROL.L-1, the first cluster in left Rolandic operculum; ROL.L-2, the second cluster in the left Rolandic operculum; ROL.R, right Rolandic operculum; INS.L, left insula; PoCG.L, left postcentral gyrus; SMG.L, left supramarginal gyrus; STG.L, left superior temporal gyrus. *A *P*-value of <0.05 after the adjustment of age and sex at scanning.

As illustrated in Figure 4, there was no significant correlation between clinical factors, i.e., age at onset and disease duration, and the FC values of the above seven thalamocortical circuits. Moreover, none of the FC values showed a significant correlation with baseline GMD or ALFF of the left thalamus. However, the FC value of each circuit had a significant positive correlation with the corresponding outflow and inflow EC values.

### Reproducibility analysis

We excluded seven patients that were identified from the prior database from the ANOVA analysis for FC and found that the brain areas displaying an intergroup difference of FC, as depicted in Supplementary Figure S3, were similar to those observed when including these patients (i.e., the clusters illustrated in Figure 1). Furthermore, considering the potential biases caused by structural and functional states, we included the GMD and ALFF of the thalamus, along with sex and age at scanning, into the ANOVA analysis and found that the brain areas displaying an intergroup difference of FC, as depicted in Supplementary Figure S4, were almost identical to those obtained by only sex and age at scanning as covariates (i.e., the clusters illustrated in Figure 1).

## Discussion

We aimed to analyze the associations between the thalamic connection and the initial ASM responses in epilepsy and found that patients exhibiting stronger functional connectivity between the thalamus and the cortex were at a higher risk for a poor response to medication.

Prior studies have reported gray matter atrophy (26, 27) or increased functional activity of the thalamus (28, 29) in epilepsy patients. Therefore, our study first estimated the baseline structural and functional features of thalami and partially confirmed the previous findings. Specifically, epilepsy patients, particularly those in the PR group, showed a trend of lower GMD and higher ALFF of each thalamus relative to the control group, although this was not significant. Then, we analyzed thalamic connectivity features and found that patients with a poor ASM response presented abnormally strengthened thalamocortical FC, accompanied by an increase in thalamic outflowing and inflowing information, compared to those with good ASM response and healthy controls. Moreover, the FC value of each thalamic circuit was positively correlated with the corresponding outflow and inflow EC values. Previous studies have reported that patients with temporal lobe epilepsy who exhibited enhanced thalamocortical functional connections were more likely to experience focal to bilateral tonic-clonic seizures (14, 15). Medication-resistant patients showed an increased within-module degree of a thalamocortical network compared to medication-responsive patients (17). A recent study has found that increased strength of the thalamocortical FC would increase the risk of seizure relapse following ASM withdrawal (18). Additionally, studies focusing on vagal nerve stimulation (19) or surgery (11, 16) have demonstrated that stronger FC of specific thalamocortical pathways is associated with postoperative seizure recurrence. Our finding was consistent with the results of prior studies. Epileptic activity is known to result from excessive synchronization of the brain network (30), and the thalamus is recognized as the central hub of the brain network with the role of promoting communication between brain areas, that is, functioning as the “synchronizer” of the brain network (10, 11). Therefore, we highlight that abnormally increased information communication between the thalamus and cortex, which facilitates brain network synchronization and ultimately promotes the emergence of epileptic activity, may be a primary mechanism of poor response to initial medication.

We found that the abnormally strengthened thalamic FC in patients with a poor ASM response involved cortical areas within the sensorimotor network, specifically the Rolandic operculum and the postcentral gyrus. This could be supported by previous studies, where patients with intractable epilepsy (31) or those experiencing persistent postoperative seizures (16) exhibited excessive FC between the thalamus and the Rolandic operculum or the postcentral gyrus. Furthermore, Tangwiriyasakul et al. found that patients with generalized genetic epilepsy had temporally stable sensorimotor synchronization from the pre-ictal to the post-ictal periods (32), and both epilepsy patients and their unaffected first-degree relatives showed higher synchrony only in the sensorimotor network compared to healthy controls (33). Yaakub et al. (34) found that both temporal lobe epilepsy patients and their asymptomatic relatives displayed a combination of increased activation and a failure of deactivation related to alpha oscillations in the sensorimotor network (34). These studies indicate that excessive synchrony and failure of deactivation in the sensorimotor network represent an inherited endophenotypic predisposition to epilepsy (33, 34). Therefore, the sensorimotor network should not be considered solely as a location where epileptiform activities occasionally originate from or spread across; instead, it should be regarded as a primary network involved in engaging large-scale brain circuits to facilitate seizure generation (33). Consequently, the increased interactions between the thalamus and the sensorimotor network would promote network synchrony, which may impede seizure control with ASM treatment.

The insula is a cytoarchitecturally complex structure (35). Relying on rich connections with other brain structures, such as extensive afferent and efferent pathways with the thalamus (36), the insula may serve as a critical hub for modulating epileptic activity. Gotman et al. reported simultaneous activation in the bilateral insulae and thalami during generalized bursts of epileptic discharges, indicating that increased thalamus-insula coupling contributes to the propagation of epileptic activity (37). Moreover, previous studies have highlighted the involvement of the thalamus-insula circuit in the progression and prognosis of epilepsy (15, 31). For instance, patients with temporal lobe epilepsy who experienced focal to bilateral tonic-clonic seizures exhibited increased thalamus-insula coupling than those experiencing only focal seizures (15). Moreover, patients with intractable epilepsy displayed abnormally increased thalamus-insula functional connectivity (31). Undoubtedly, our consistent finding of increased FC between the thalamus and the insula in patients with poor ASM response provides new evidence on the clinical implications of the thalamus-insula circuit for assessing the initial ASM responses in epilepsy.

This study also found that patients with a poor ASM response had strengthened thalamic FC with the supramarginal and the superior temporal gyrus, which was consistent with prior studies reporting increased FC between the thalamus and these cortical areas in patients with refractory epilepsy (31) or persistent postoperative seizures (16). The thalamus serves as an integrative hub for multiple cognitive processes by mediating information communication within and between functional cortical networks that are putatively associated with distinct cognitive functions (10). The supramarginal gyrus is a major part of the inferior parietal lobe and has been found to be involved in external/perceptual information processing, memory encoding (38), and phonological word decision (39). The superior temporal gyrus, containing part of the auditory cortex and Wernicke's area, has been reported to provide an interface for transforming external acoustic speech signals into internal representations of words, which is critical for speech perception and language comprehension (40). Therefore, in addition to their involvement in ASM response, these aberrant thalamocortical circuits may be related to cognitive comorbidities/complications of epilepsy, which are quite common even in newly diagnosed epilepsy patients (41). Additionally, cognitive function, e.g., memory performance, has been identified as a predictor of refractory epilepsy (42), which is likely due to a shared pathogenetic mechanism between cognitive disorders and epilepsy (43). Thus, our findings indicate a possible mechanism underlying the association between cognitive impairment and seizure control with ASM treatment.

Intriguingly, we found that the cortical areas exhibiting excessive functional connectivity with the left thalamus, which increased the risk of a poor ASM response, were mainly located in the left hemisphere. Note that no functional connectivity of the right thalamus was associated with a poor ASM response. Consistent with our finding, a prior study revealed that the abnormalities in thalamocortical white matter fiber tracts, as measured by radial diffusivity, were concentrated in the left hemisphere of patients with medication-resistant generalized epilepsy (44). Additionally, patients with temporal lobe epilepsy exhibited more cortical atrophy and white matter fiber damage in the left hemisphere than in the right hemisphere (45). This phenomenon may be related to the asymmetry of the brain network. The left hemisphere has been found to mature later and more slowly than the right hemisphere (46), rendering it more vulnerable to seizure attacks (45). Moreover, individuals who are left-hemisphere dominant for language have been reported to exhibit more extensive connectivity in the left hemisphere than in the right hemisphere (47). Thus, the functional connectivity of the left hemisphere in patients with right-handedness, as observed in our study, will display more distinct responses to seizure progress than that of the right hemisphere. However, we cannot rule out a less favorable explanation that some functional thalamocortical connectivity in the right hemisphere also differed between groups, but our study's limited sample size was insufficiently powered to detect it.

Some caution needs to be exercised when interpreting the findings of this study. First, the relative sample size, as mentioned above, may challenge the reliability of our findings to some extent. Second, while both epilepsy etiology and seizure type showed no difference between the groups, their potential effects on brain networks should not be entirely disregarded. Future studies with larger sample sizes are necessary to validate our findings in patients with a single epilepsy etiology or seizure type. Third, this study provided information on the laterality of epileptic discharges but could not accurately identify the epileptic syndrome for each case since most patients had no epileptic episodes during electroencephalography monitoring. Fourth, disease duration before ASM administration varied among patients. However, we conjectured that this heterogeneity had a limited effect on our main findings, as no correlation was found between disease duration and targeted functional connectivity. Finally, previous research has indicated a bidirectional relationship between epilepsy, cognitive dysfunction, and psychiatric disease, with a probable shared network mechanism (41). Therefore, the rough assessment of comorbidities in this study may have inadvertently overlooked subtle cognitive decline or psychiatric disorders, resulting in the concealment of the cognitive and psychiatric implications of our findings. Despite these limitations, as one of the first to explore thalamic connection features associated with the initial ASM response in epilepsy, this study provided relevant insights into the network mechanism underlying seizure control with ASM treatment.

## Conclusion

Our study demonstrated that epilepsy patients with excessively strong functional connectivity between the thalamus and the cortical areas spanning the frontal, parietal, and temporal lobes might be more likely to respond poorly to initial antiseizure medication. These reinforced recursive thalamocortical circuits tended to be driven by a combination of outflowing information from the thalamus to the cortex and inflowing information from the cortex to the thalamus.

## Data availability statement

The raw data supporting the conclusions of this article will be made available by the authors, without undue reservation.

## Ethics statement

The studies involving human participants were reviewed and approved by the Ethics Committee of West China Hospital of Sichuan University. Written informed consent to participate in this study was provided by the participants' legal guardian/next of kin.

## Author contributions

JZ and YC designed the study, interpreted the results, and drafted the manuscript. GT and HW recruited participants and collected data. JZ, GT, and HW performed data preprocessing and analysis. All authors revised the manuscript and approved the submitted version.
