# Supplementary material for: Excessively increased thalamocortical connectivity and poor initial antiseizure medication response in epilepsy patients
Source: Front Neurol. 2023 Jun 16;14:1153563. doi: 10.3389/fneur.2023.1153563 (PMC10312096; doi:10.3389/fneur.2023.1153563)
Supplement: Supplementary file 1 [file Data_Sheet_1.docx]

Supplementary materials

FIGURE S1 Flow diagram of inclusion procedure of patients


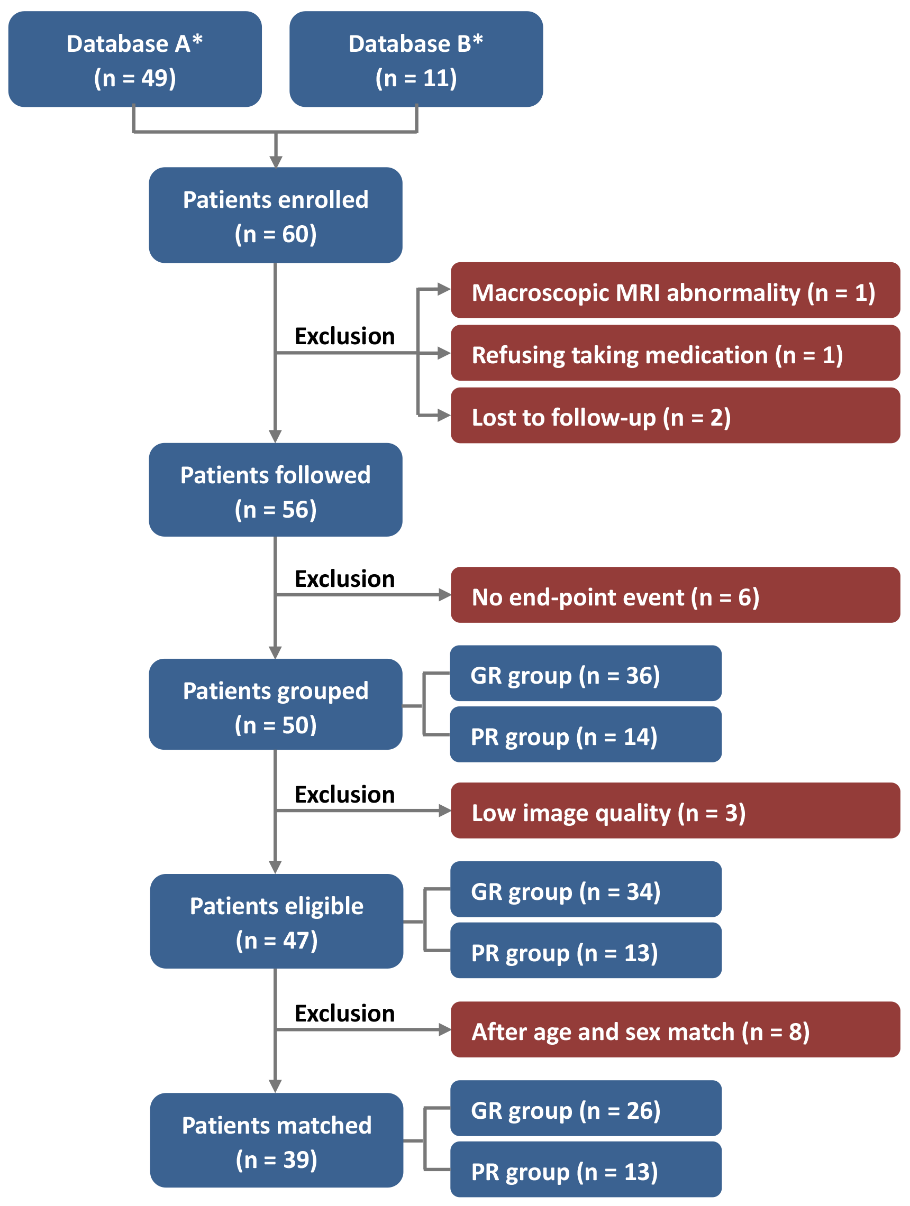


Abbreviations: MRI, magnetic resonance imaging; GR group, patients with good response to antiseizure medication; PR group, patients with poor response to antiseizure medication.

* Database A was established to prospectively enroll patients with newly diagnosed epilepsy, with 49 patients enrolled from October 2019 to December 2021 included in this study; while Database B was established to prospectively enroll patients with first epileptic seizure from September 2016 to December 2019, with 11 patients who met the diagnosis of new-onset epilepsy included in this study.

FIGURE S2 Procedures from functional connectivity to effective connectivity


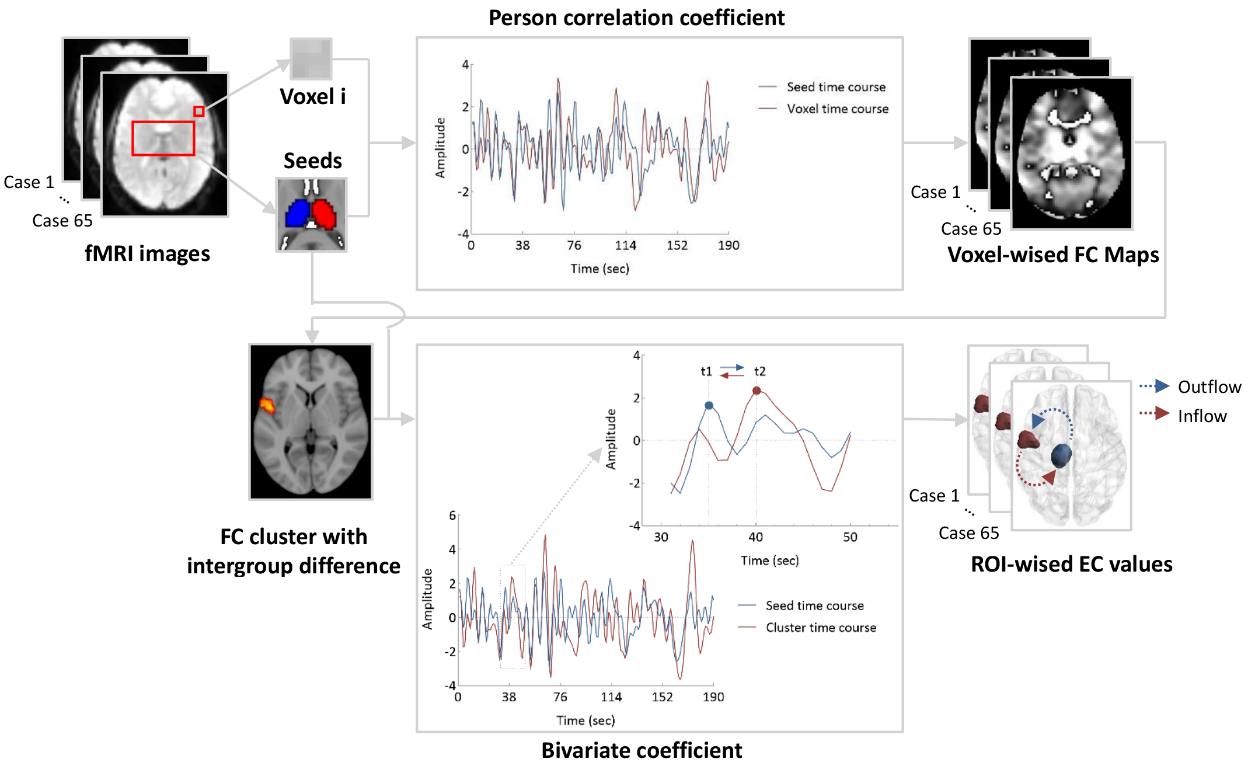


Abbreviations: EC, effective connectivity; FC, functional connectivity; fMRI, functional magnetic resonance imaging; ROI, region of interest.

FIGURE S3 Reproducibility analysis for the intergroup differences in functional connectivity of left thalamus according to sample size of patients


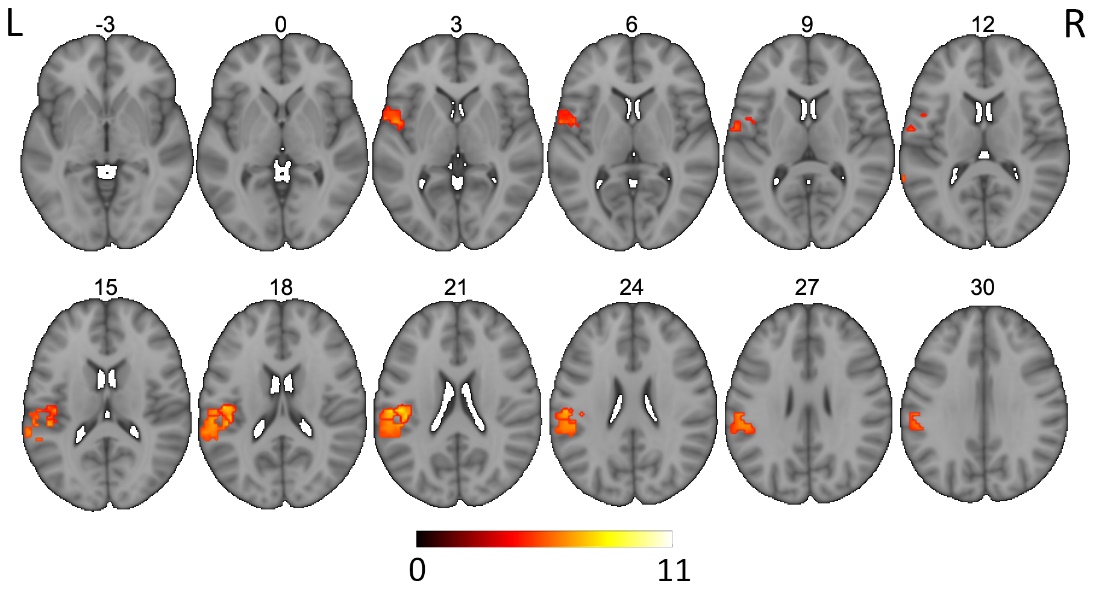


Showing clusters surviving the False Discovery Rate correction with *P* < 0.05 and cluster size > 30 voxels; comparing between the Control (n = 26), GR group (i.e., patients with good response, n = 21) and PR group (i.e., patients with poor response, n = 11), with the 7 patients recruited in prior database, including 5 in the GR group and 2 in the PR group, excluded; sex and age at scanning included as covariates.

FIGURE S4 Reproducibility analysis for the intergroup differences in functional connectivity of left thalamus according to covariates


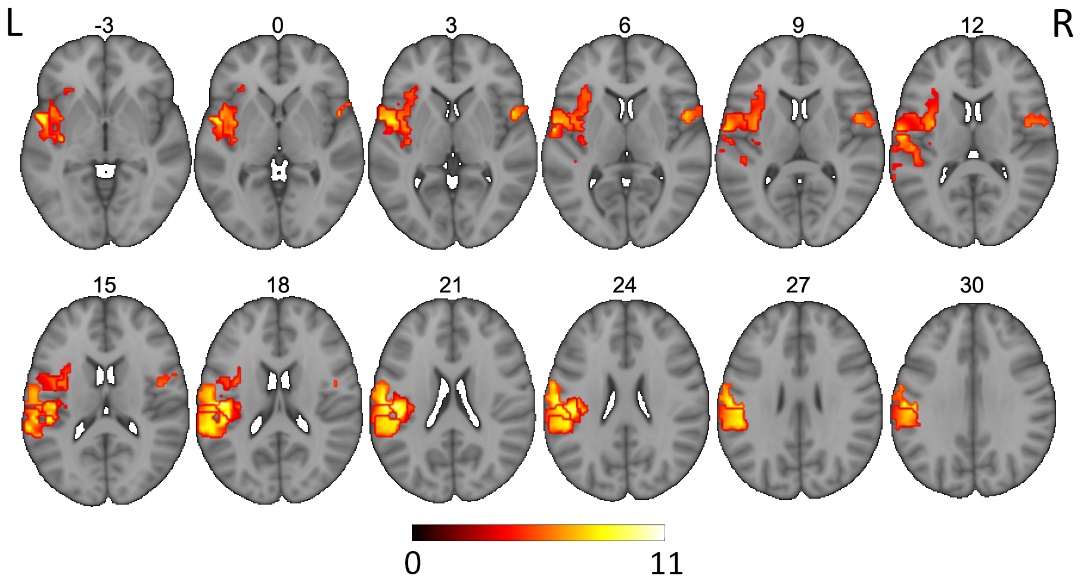


Showing clusters surviving the False Discovery Rate correction with *P* < 0.05 and cluster size > 30 voxels; comparing between the Control (n = 26), GR group (i.e., patients with good response, n = 26) and PR group (i.e., patients with poor response, n = 13), with sex, age at scanning and the left thalamic GMD (i.e., grey matter density) and ALFF (i.e., amplitude of low-frequency fluctuation) included as covariates.

Table S1 Demographic and clinical characteristics between patients included at different times

| Variables | EC group  （n = 7） | LC group  （n = 32） | *P value* |
| --- | --- | --- | --- |
| Female, n (%) | 3 (42.9%) | 15 (46.9%) | 1.00 |
| Age at scanning, y | 29.57 ± 11.77 | 33.13 ± 12.46 | 0.50 |
| Age at onset, y | 29.57 ± 11.77 | 29.38 ± 14.17 | 0.97 |
| Disease duration, m * | 0.33 (0.23 - 0.47) | 21.50 (6.25 – 74.25) | < 0.001 |
| Follow-up period, m * | 45.00 (7.00 - 53.00) | 25.00 (7.25 – 32.00) | 0.02 |
| Etiology, n (%) |  |  | 0.33 |
| Genetic | 1 (14.3%) | 1 (3.1%) |  |
| Unknown | 6 (85.7%) | 31 (96.9%) |  |
| Seizure type, n (%) |  |  | 0.004 |
| Focal onset | 2 (28.6%) | 27 (84.4%) |  |
| Generalized onset | 1 (14.3%) | 3 (9.4%) |  |
| Unknown onset | 4 (57.1%) | 2 (6.3%) |  |
| Seizure frequency, n (%) |  |  | 0.16 |
| ≤ ten | 7 (100.0%) | 21 (65.6%) |  |
| > ten | 0 (0.0%) | 11 (34.4%) |  |
| Discharge laterality, n (%) |  |  | 0.35 |
| No discharge | 5 (71.4%) | 10 (31.3%) |  |
| Left discharge | 0 (0.0%) | 4 (12.5%) |  |
| Right discharge | 1 (14.3%) | 7 (21.9%) |  |
| Bilateral discharge | 1 (14.3%) | 11 (34.4%) |  |
| Medication type, n (%) |  |  | 0.18 |
| Monotherapy of levetiracetam | 4 (57.1%) | 22 (68.8%) |  |
| Monotherapy of valproate | 0 (0.0%) | 1 (3.1%) |  |
| Monotherapy of oxcarbazepine | 0 (0.0%) | 5 (15.6%) |  |
| Monotherapy of topiramate | 1 (14.3%) | 0 (0.0%) |  |
| Polytherapy | 2 (28.6%) | 4 (12.5%) |  |
| Response to medication |  |  | 1.00 |
| Good response | 5 (71.4%) | 21 (65.6%) |  |
| Poor response | 2 (28.6%) | 11 (34.4%) |  |

Abbreviations: EC group, patients recruited early; LC group, patients recruited later; y, year; m, month. * Presented as median with interquartile range, and compared using Mann-Whitney U-test.
